# New diagnostic strategy for multiple myeloma: A review

**DOI:** 10.1097/MD.0000000000036660

**Published:** 2023-12-29

**Authors:** Ligong Xu, Shuang Wu

**Affiliations:** a Department of Radiology, The First Affiliated Hospital Zhejiang University School of Medicine, Hangzhou, China; b Department of Nuclear Medicine and PET Center, The Second Affiliated Hospital Zhejiang University School of Medicine, Hangzhou, China.

**Keywords:** clinical features, imaging, laboratory examination, multiple myeloma

## Abstract

Multiple myeloma (MM) is the second most prevalent hematological malignancy and is distinguished by the aberrant proliferation of monoclonal plasma cells inside the bone marrow and production of M-protein. This condition frequently results in bone deterioration, acute kidney damage, anemia, and hypercalcemia. However, the clinical manifestations and accompanying symptoms of MM vary and may change as the condition evolves. Therefore, diagnosis of MM is difficult. At present, the confirmation of MM diagnosis necessitates the use of bone marrow biopsy, a procedure that is both invasive and challenging for assessing dynamic alterations in the disease. The integration of laboratory testing technologies with imaging technology has the potential to enhance the diagnostic effectiveness and provide a thorough evaluation of disease progression and prognosis in patients with MM. All the examination methods have advantages and disadvantages. Therefore, diagnosis is determined by the application of clinical characteristics, serological tests, and imaging investigations.

## 1. Introduction

Multiple myeloma (MM) is the second most common hematological malignancy^[1]^ and is characterized by the abnormal growth of monoclonal plasma cells in the bone marrow that produce M proteins. This condition often leads to bone destruction, acute renal injury, anemia^[2]^ and hypercalcemia.^[3]^ While bone damage is the most common indication for MM,^[4]^ it is detected in approximately 2-thirds of patients upon initial diagnosis and eventually develops in nearly all patients as the illness advances. The degradation of bone tissue has a detrimental effect on the overall well-being of individuals and is a significant contributor to both illness and death.^[5]^ Confirmation of the diagnosis of multiple myeloma necessitates the use of bone marrow biopsy and serum immunoelectrophoresis^[6]^ because the sensitivity of laboratory indicators and imaging manifestations in this context is very limited. Therefore, diagnosis is determined by clinical characteristics, serological tests, and imaging investigations.

## 2. Clinical features

Under these pathological conditions, various tissues and organs experience direct infiltration by numerous clonal plasma cells that originate from the bone marrow. Secretion of M proteins by these cells leads to diverse clinical symptoms.^[7]^ Among these symptoms, the most notable include bone pain, bone deformation, pathological fracture, anemia or hemorrhage, hypercalcemia, and renal function impairment. The presence of myeloma cells in the body leads to bone pain, deformation, and pathological fractures. This is attributed to the production of osteoclast-activating factors by myeloma cells,^[8]^ which subsequently stimulates osteoclast activity.^[9]^ As a result, bone disintegration and destruction occur. Potential outcomes include bone pain, bone deformation, and pathological fracture.^[10]^ The primary manifestation is skeletal discomfort, with a higher incidence in the lumbosacral region, followed by the thoracic spine, ribs, and bones of the lower extremities. Pathological fractures manifest due to the breakdown of bone by tumor cells, often presenting with several concurrent fractures. The defining appearance of myeloma cell infiltration is the formation of “bead-like nodules” at the intersection of the chest, ribs, and clavicle. Anemia or hemorrhage: Approximately 90% of individuals exhibit varying degrees of anemia,^[1,11]^ with some experiencing it as their initial symptom. The manifestation of anemia is mild during the initial phases^[12]^ but becomes more severe in later stages.^[13]^ During the advanced phase, thrombocytopenia may manifest, leading to hemorrhagic signs. The occurrence of bleeding in the skin and mucous membranes is more widespread; however, in extreme cases, bleeding can also manifest in visceral organs and the brain. The hypercalcemia syndrome^[14]^ is characterized by a decrease in calcium clearance caused by osteoclast-mediated bone reabsorption and a reduction in the glomerular filtration rate. Renal function impairment can manifest as proteinuria, cylinduria, and acute or chronic renal failure, among other symptoms.^[14]^ The causes of peripheral protein deposition, tubular urine, and acute and chronic renal failure are as follows: several factors contribute to the occurrence of these conditions. These factors include the deposition of peripheral proteins in the proximal renal tubules, development of polyuria or oliguria due to hypercalcemia, and the excessive presence of uric acid. Extramedullary infiltration refers to infiltration of cells or tissues outside the bone marrow. The aforementioned ailments are distinguished by enlargement of the liver, spleen, and lymph nodes. Additionally, if the nervous system is affected, it may lead to limb paralysis, lethargy, coma, diplopia, and other visual impairments including blindness and loss of visual acuity.

## 3. Laboratory examination

Individuals diagnosed with multiple myeloma exhibit anomalous proliferation of myeloma plasma cells, atypical manifestation of serum immunoglobulin and inflammatory markers, heightened levels of blood calcium, and unaltered blood phosphorus levels. Moreover, it is worth noting that proteinuria is observed in over 90% of patients, with around 50% of them exhibiting periplasmic protein. Chromosomal aberrations can potentially serve as an indication for multiple myeloma.

(1) Blood examination: Most patients had orthochromatic normocytic anemia, characterized by the presence of the money-string phenomenon in the red blood cells shown on the peripheral blood film. The blood sedimentation rates were elevated, occasionally exceeding 100 mm/h. White blood cell and platelet counts often remain within the normal range. As a result, the viscosity of the plasma increased. Approximately 80% of serum protein electrophoresis exhibits spikes and M-protein bands as a result of monoclonal immunoglobulins, and the type of immunoglobulin can be determined by immunological techniques. Frequently, there is a reduction in the serum albumin levels. The levels of serum 2-microglobulin increased, and the extent of this increase correlated with the number of tumor cells present in the organism. Hypercalcemia affects approximately 10% of patients with an initial diagnosis. Elevated blood urea nitrogen, creatinine, and uric acid levels may also be observed. Typically, serum alkaline phosphatase levels remain within the normal range; however, they may be elevated during fracture healing or in instances of hepatic amyloidosis. In addition, several researchers have identified associations between MM and distinct biomarkers including hepcidin 25, growth differentiation factor 15 (GDF15), soluble transferrin receptor (sTfR), and zonulin. Elevated concentration of hepcidin-25 was reported with the advancement of MM. In addition, it was also related with poor response to MM treatment, revealing its potential being as survival predictor of MM.^[15]^ Likewise, lower expression of GDF 15 was found in patients those received autologous stem cell transplantation compared to control groups, which indicated GDF 15 was able to predict response to treatment . Nevertheless, the predictive effect of GDF 15 on survival of MM patients is still in debate, because current research results were different.^[16,17]^ As for sTfR, it is thought that sTfR measurement may be the most important biomarker to uncover the etiology of anemia in MM compared to more established biomarkers, such as serum ferritin and C-reactive protein in theory.

Direct reports about sTfR and MM are rare. One or more of these biomarkers are novel indicators of iron regulation and have the potential to serve as prognostic indicators in patients with multiple myeloma and renal insufficiency in the foreseeable future.^[18–21]^ Hepcidin is a peptide hormone consisting of 25 amino acids that is synthesized in the liver and plays a role in the negative regulation of iron metabolism. The protein in question is responsible for governing the transfer of iron from enterocytes to the bloodstream, regulating the transfer of iron from hepatocytes that store iron, releasing iron from macrophages, and aiding in the transportation of iron through the placenta. Hepcidin synthesis is induced by increased plasma iron concentrations, a process that may be modulated by iron storage and proinflammatory cytokines.

Despite the increased activity of erythropoietin (EPO), hepcidin exerts inhibitory effects. Moreover, GDF15 is believed to belong to the transforming growth factor β superfamily, yet it has also been shown to exhibit significant expression in erythroblasts, suggesting its potential involvement in the control of erythroid lineage profiles.^[22]^ The ability of GDF15 to facilitate the progression of anemia may stem from its capacity to regulate hepcidin expression. An association between GDF-15 levels and inadequate treatment response in MM has been established. Furthermore, the measurement of GDF-15 holds clinical promise for predicting disease progression and outcome. This predictive capability may also have an indirect impact on the evaluation of anemia, as a reduction in malignant cell infiltration in the bone marrow could potentially enhance hematopoiesis.^[16,20]^ Additionally, Katodritou et al^[23]^ provided further support for this concept by examining a cohort of 26 patients who were newly diagnosed with MM and were anemic (hemoglobin level < 10.5g/dL). The authors found that the predictive value of ferritin index and hypochromic erythrocytes was significant in this patient population. The purpose of this index is to identify individuals who are likely to benefit from recombinant EPO and to determine if they have a functional iron deficiency that would necessitate iron supplementation during the diagnosis and early phases of treatment. In addition, a recent study identified Pituitary adenylate cyclase-activating polypeptide as a novel noninvasive biomarker of MM,^[24]^ and higher concentration of Pituitary adenylate cyclase-activating polypeptide levels revealed longer survival and better response to the clinical treatment. Collectively, more and more new serum biomarkers are exploring and meanwhile mounting evidence are still needed to further demonstrate the efficiency of those biomarkers.

(2) Urine analysis: Urine proteins and tubulin, mostly deposited by light chains, exhibited frequent positivity. The occurrence of significant proteinuria in myeloma is rare, and its presence suggests the coexistence of amyloidosis and light chain deposition lesions. The presence of urinary periplasmic protein, specifically monoclonal light chains that are able to traverse glomerular filtration, may be reliably detected and quantified in concentrated 24-hour urine samples using immunoelectrophoresis or gel immunoelectrophoresis. However, the detection rate decreases to just 40–50% when heating was employed. The κ-to-λ ratio of the urinary light chain is 2:1. The assessment of monoclonal free light chains in patients with MM has traditionally been monitored using electrophoretic measures of Bence Jones protein in 24-hour urine samples. However, there has been considerable debate over the suitability of this method for evaluating free light chain response. The efficacy of urinalysis in monitoring patients with MM is limited by factors such as limited analytical sensitivity, influence of renal metabolism, and inadequate supply of urine samples.^[25]^

(3) Bone marrow analysis: The occurrence and advancement of hematological tumors are followed by changes in the surrounding tissue microenvironment,^[26,27]^ including both hematopoietic and non-hematopoietic cells, along with non-cellular constituents.^[28]^ Bone marrow examination revealed the presence of proliferating myeloma cells.^[29]^ Diagnosis of myeloma may require a significant number of bone marrow punctures because of the possible presence of myeloma cells that might be widely or locally distributed in a patchy manner inside the bone marrow. Bone marrow biopsy can augment the detection rate. M-proteins in myeloma cells were detected using immunoenzymatic labeling methods.^[30]^ The potential of employing the plasma cell tritium labeling index lies in its ability to distinguish the presence of DNA synthesis inside myeloma cells, thereby functioning as a signal for the proliferation of these cells in the context of myeloma.

## 4. Imaging characteristics

The detection of bone and bone marrow lesions is of significant importance in the domain of multiple myeloma research, as it serves as a crucial factor in identifying the most suitable therapeutic approach. The initiation of treatment for individuals with myeloma bone disease is of utmost importance, particularly in cases where imaging alterations are observed, even in the absence of clinical symptoms.^[31,32]^ This phenomenon can be attributed to the increased susceptibility of these individuals to accelerated development of illness. Various imaging modalities such as X-rays, computed tomography (CT), magnetic resonance imaging (MRI), and positron emission tomography (PET) play crucial roles in the diagnosis, staging, and assessment of therapeutic efficacy in patients with multiple myeloma. It is crucial to first recognize lytic bone lesions^[33]^ as they act as an indication of disease-related harm to essential organs and are commonly used for the identification of MM and to assess the necessity of prompt medical intervention. Furthermore, imaging modalities can proficiently identify regions affected by extramedullary disease, a prognostic indicator linked to unfavorable results. Moreover, imaging techniques play a crucial role in differentiating solitary plasmacytoma (SP) from MM and in assessing the probability of progression from smoldering MM to an active state of the illness. In the context of MM, the application of imaging modalities plays a pivotal role in the initial diagnosis of recurrence, as well as in the detection of sites of bone degradation that may be prone to pathological fractures or neurological complications. Functional imaging approaches provide the ability to enhance the precision of assessing the extent of therapy response, especially in individuals who have been diagnosed with non-secretory MM and have a normal serum-free light chain ratio. Furthermore, these methodologies have a more expansive function in the assessment of negative minimal residual disease (MRD).

(1) X-rays: The conventional approach utilizing X-ray imaging is a prevalent technique employed to identify bone-infiltrating lesions in patients with MM.^[34]^ This method is cost-effective and extensively utilized. X-ray imaging is mostly utilized for the identification of long bones in the limbs and cranial abnormalities, enabling visual representation of alterations in skeletal structure.^[6]^ Most patients exhibit pronounced symptoms of bone alteration characterized by the presence of round-shaped translucent areas as the primary manifestation. These areas vary significantly in size and have clear boundaries. In addition, most patients display bone destruction in the form of puncture-like, honeycomb-like, or worm-eating patterns. Some patients also present with paravertebral soft tissue masses, interruptions in the bone cortex, and other related manifestations. Several investigations have indicated a strong correlation between bone lesions and tumor burden as well as prognosis. Consequently, alterations observed in X-ray images have been incorporated into clinical staging systems. Specifically, individuals exhibiting rounded osteolytic lesion image presentations were categorized as having D-S stage III illness.^[35]^ However, X-ray examinations have certain limitations. First, the sensitivity of conventional X-ray examinations is restricted by their ability to detect certain conditions. Additionally, the false-negative rate ranges from 30% to 70%, which can result in underdiagnosis of MM lesions. What more, the conventional X-ray examination process necessitates multiple repositioning of the patients, potentially causing inconvenience. Finally, X-ray examinations were unable to detect extramedullary disease nor cord involvement with MM. Based on these shortcomings, X-ray examinations were gradually replaced by other more sensitive techniques like CT and MRI.

(2) CT: Whole-body low-dose computed tomography (WBLDCT) is a frequently used modality for assessing the entire skeletal system in cases of multiple myeloma. The sensitivity and specificity of the WBLDCT were 70% and 90%, respectively. It has been found to be more effective than traditional radiography in assessing fracture risk, reducing the examination time, and identifying extramedullary abnormalities. Hence, the European Myeloma Society has proposed the substitution of traditional X-rays with WBLDCT as the prevailing imaging modality, according to their recommendations. Furthermore, the European Myeloma Network and the European Society for Medical Oncology endorsed the use of WBLDCT as the preferred method for evaluating osteolytic damage in myeloma.^[6]^ Plain scanning can effectively visualize bone destruction and tumor involvement in the bone and soft tissues, and provides a clear depiction of the tumor relationship with neighboring structures. Additionally, it can detect tumor invasion into the medullary cavity, which is characterized by the presence of fat in low-density bone marrow. Contrast-enhanced scanning enhances the visualization of the parenchymal portion of the tumor, facilitating differentiation of the tumor from the surrounding tissues. Furthermore, it enables visualization of the relationship between the tumor and blood vessels, which can guide surgical treatment decisions. Several investigations have demonstrated that WBLDCT can not only evaluate isolated osteolytic lesions but also detect widespread bone marrow involvement by analyzing the attenuation pattern of the surrounding medulla.^[36]^

In addition to WBLDCT, dual-energy CT (DECT), especially third-generation dual-energy CT, enables low-effective-dose scanning with higher sensitivity than conventional CT examination methods and comparable diagnostic performance to that of MRI.^[37]^ The utilization of virtual decalcification imaging technology in dual-energy CT enables further quantification of the bone marrow attenuation value by calculating the bone marrow density following the elimination of cancellous bone trabeculae. This process aids in the precise evaluation of response to MM treatment. Furthermore, it exhibits a notable degree of sensitivity for evaluating the infiltration pattern of MM inside the bone marrow, thereby enhancing the identification of high-grade diffuse infiltrating lesions.^[38]^

Advances in AI technology have enhanced the diagnostic efficacy of MM, particularly in the realm of imaging and AI integration. In a study conducted by Francis et al,^[39]^ it was demonstrated that the utilization of photon counting detector (PCD) computed tomography, coupled with deep learning noise reduction techniques, has the potential to enhance the spatial resolution of multiple myeloma in comparison with energy-integrated detector (EID) CT. In a study employing a blinded evaluation, it was observed that PCD CT, when compared to EID CT, exhibited enhanced visualization of cytopathic lesions, intramedullary lesions, fatty metaplasia, and pathological fractures in 2 mm images. Furthermore, for the comprehensive assessment of all 4 pathological abnormalities, the implementation of convolutional neural network denoising in 0.6 mm PCD CT images also demonstrated improvement.

(3) MRI: MRI possesses several notable benefits, including exceptional soft-tissue resolution, multiparameter and multisequence imaging, and radiation-free nature. Moreover, MRI enables both qualitative and quantitative diagnosis of diverse pathophysiological alterations within the body. Consequently, it is the preferred method for identifying and assessing bone marrow infiltration, particularly in cases involving the spine and pelvis. Multiple investigations^[40–43]^ have demonstrated that MRI has greater efficacy in the detection of localized lesions than whole-body X-ray imaging. Additionally, MRI demonstrates diagnostic performance equivalent to that of CT and PET/CT. Nevertheless, the drawbacks of this technique include a relatively sluggish imaging speed, a lack of sensitivity towards areas and abnormalities with a lower concentration of hydrogen protons (such as those with higher amounts of calcium), and high cost. Furthermore, it is contraindicated in patients with claustrophobia or metal implants.

Diffusion-weighted imaging (DWI) is a magnetic resonance imaging method that uses the random movement of water molecules, known as Brownian motion, to generate images. This sequence is very sensitive for detecting bone marrow lesions and enables quantitative assessment of bone marrow infiltration without the need for invasive procedures. Quantification of the apparent diffusion coefficient (ADC) is important for diffusion-weighted magnetic resonance imaging (DW-MRI). This value serves as an indicator of the nucleoplasmic ratio, cell count, and ability of water molecules to diffuse. The utilization of ADC mapping, which is obtained from DWI, enables differentiation of bone marrow alterations in active myeloma from those observed during remission.^[44]^ This process provides therapeutically significant information regarding tumor survivability. Nevertheless, the DWI technique has a deficiency in terms of specificity and, hence, necessitates the integration of additional imaging modalities to enhance its diagnostic accuracy. Multiple studies have demonstrated a positive correlation between the magnitude of alteration in ADC values and the level of malignancy exhibited by certain tumors. Within the cohort of patients with MM experiencing remission, the comprehensive bone marrow high-b-value image exhibited a diminished signal. Additionally, the average ADC value initially demonstrated an elevation, whereas no notable alterations were observed on T1-weighted imaging or T2-weighted imaging at this juncture. These findings suggest that modification of the DWI signal may serve as a valuable indicator for early evaluation of treatment response. In their study, Messiou et al^[45]^ observed a decline in ADC values at 20 weeks after chemotherapy. This decline is likely attributable to the interplay of multiple factors, including remodeling of deceased tumor cells within the tissue architecture, clearance by macrophages, and delayed restoration of the adipose component. Furthermore, the utilization of quantitative analysis to evaluate the ADC values of identified lesions using whole-body magnetic resonance imaging (WB-MRI) in patients with MM undergoing early therapy might contribute to the evaluation of clinical outcomes. Moreover, Koutoulidis et al observed that diffuse patterns of MM infiltration exhibited a higher apparent diffusion coefficient (ADC) on imaging than localized lesions. They further determined that an ADC value of 548 µmm2/s demonstrated a sensitivity of 100% and specificity of 98% in distinguishing a diffuse pattern of myeloma infiltration from normal marrow.^[46]^

The utilization of the Dixon technique for magnetic resonance imaging water-fat separation has significant clinical significance in the assessment of MM, including its diagnosis, therapy, and prognosis evaluation. The water-fat separation imaging approach capitalizes on the disparity in resonance frequencies between water and fat, enabling the capture of 2 sets of data through manipulation of the echo duration. The initial acquisition aligned the transverse magnetization intensity vectors of water and fat, resulting in a combined image of both substances. Subsequent acquisition induces opposite phases of water and fat, generating a different image. The lipid and water components can be separated by performing arithmetic operations on these images, thereby achieving water–lipid separation. However, the effectiveness of this approach is affected by the strength of the magnetic field, which impedes the complete separation of the water and fat. Consequently, the water-fat interface structure becomes indistinct, and the signal-to-noise ratio of the image decreases. Owing to ongoing advancements in technology, the initial approach for acquisition has undergone modifications, resulting in the adoption of the 3-point method for asymmetric echo water-fat separation imaging. This method was further refined through iterative decomposition of water and fat with echo asymmetry and least-squares estimation quantification sequence, commonly referred to as IDEAL-IQ. This technology not only reduces the duration of the scanning process but also guarantees the precise separation of water and fat components, regardless of the water-to-fat ratio. Recently, there has been development in the use of gradient-echo-based Dixon MRI as a tool for quantifying fat. This methodology includes the production of 4 distinct types of images: in-phase (IP), out-of-phase (OP), water-only (WO), and fat-only (FO).^[47–49]^ Furthermore, research has demonstrated that T2 Dixon fat-only Dixon images exhibit greater efficacy in detecting lesions in multiple myeloma than in-phase images alone.^[50]^

(4) PET: The International Myeloma Work Group suggests that PET/CT be employed to evaluate the distribution and activity level of MM as well as to analyze the physiological, biochemical, pathological, metabolic, and other biological features of tumor cells in tissues. This approach holds significant clinical importance in terms of MM grading and staging as well as in guiding personalized treatment.^[51]^ ^18^F-FDG, a glucose analog, is the most frequently used imaging agent for PET/CT. Its distribution inside the body can provide insights into the glucose metabolism of tissue cells, thereby indirectly indicating the proliferation and differentiation of certain tumor cells. Upon intravenous injection, glucose transport proteins facilitate the transport of ^18^F-FDG across the cell membrane. Once within the cell, it undergoes catalysis by hexokinase, resulting in the production of 1F-FDG-6-PO4.1F-FDG-6-PO4, which exhibits limited metabolic activity owing to its distinct structural composition compared to glucose. Through the action of glucose phosphatase, it undergoes reconversion to 1F-FDG-6-PO4, which serves as a glucose analog of ^18^F-FDG. Subsequently, it enters the interstitial space of tissues via glucose transporter proteins. The increased presence of cancer cells is linked to enhanced glycolytic activity, which is defined by raised levels of glucose transporter proteins, namely glucose transporter proteins 1 and 3. Additionally, hexokinase expression was upregulated, and glucose phosphorylase expression was downregulated. These molecular alterations collectively contribute to the substantial buildup of 1F-FDG inside tumor cells. PET/CT scanning can provide information on the in vivo distribution of 1F-FDG, which allows the identification of tumor foci in a proliferative state. This includes the detection of lymph nodes and distant metastatic foci as well as localization of the tumor foci. The utilization of PET/CT to examine the in vivo distribution of ^18^F-FDG can provide comprehensive insights into several parameters related to active tumor foci, including their spatial position, shape, dimensions, abundance, and proximity to adjacent tissues. This imaging technique can also provide valuable information regarding lymph node and distant metastases. The standardized uptake value and standardized uptake value lean body mass (SUL) are significant semiquantitative measures used in PET/CT imaging. These measures enable quantification of the extent of ^18^F-FDG uptake by a lesion, thus providing insight into the metabolic activity of a particular location. The measurement of ^18^F-FDG uptake in a lesion allows the assessment of metabolic functional activity in a particular location. The integration of anatomical structure and functional metabolism in this examination allows for the assessment of systemic pathophysiological alterations at the cellular and molecular levels. This comprehensive approach aids in objective quantitative interpretation of PET/CT images.

PET-CT demonstrates superiority over conventional skeletal X-rays in the detection of bone marrow infiltration and localized lesions while exhibiting equivalent performance to MRI in this regard.^[52]^ The suitability of PET/CT for assessing the survivability of localized lesions and the high sensitivity of MRI for detecting diffuse bone marrow involvement has been established in previous studies.^[51,53]^ The radiopharmaceutical ^18^F-FDG is commonly used in positrons, and PET/CT imaging demonstrates a notable level of efficacy in the identification of MM.^[54]^ This imaging technique can reveal various types of bone lesions such as those that cause bone destruction, diffuse bone marrow involvement, and extramedullary lesions. The sensitivity and specificity of PET/CT imaging for myeloma detection are reported to be 85% and 92%, respectively.^[6]^ Consequently, this imaging modality serves as a valuable tool for clinical monitoring and follow-up of patients with MM. Nevertheless, it should be noted that the sensitivity of 18FDG PET/CT for detecting localized lesions in MM is higher than that of WB-MRI, with an approximate estimation of 75%. Moreover, in the context of prognostication and therapy of patients with MM, 18FDG PET/CT has greater potential than WB-MRI for informing clinical decision-making.^[55,56]^

PET/MRI combines the morphological information provided by MRI with the metabolic and functional metabolic profiles provided by PET imaging, enabling the detection of myeloma-infiltrating bone marrow lesions and the assessment of prognosis and response to treatment. 18FDG PET/MRI has been reported to have a higher lesion detection rate than 18FDG PET/CT in the assessment of skeletal lesions.^[55]^ In addition, 18FDG PET/MRI increases the visibility of focal MM lesions at diagnosis and initial staging, and localizes residual disease activity after treatment.^[57]^

Compared with PET/CT, PET/MRI is more sensitive in MM staging. More than this, PET/MRI exerts better optimal spatial resolution and can detect even very small lesions or diffuse bone marrow infiltration. Further, PET/MRI could integrate the information about bone marrow cellularity and vascularization, and metabolic activity, allowing a one-stop-shop examination.^[58,59]^ In the contrast, PET/CT shows much better prognostic value at both baseline and before transplantation . Currently, hybrid PET/MRI imaging is regarded as the optimal imaging method for MM, and more evidence of clinical evidence-based medicine is needed to help us to decide how to combine these imaging techniques or which one applied in the early or advanced stage of MM.

## 5. Conclusions

The utilization of advanced testing methodologies and the emergence of cutting-edge imaging technologies have facilitated the application of novel diagnostic tests and imaging techniques in the detection and diagnosis of MM as well as in the prognostic assessment of the disease. Various combinations of different assays have been created or utilized in the field of MM to enhance the provision of comprehensive information for guiding not only the processes of diagnosis and staging, but also the assessment of treatment results. The use of these methodologies has resulted in enhanced diagnostic specificity and sensitivity in MM, leading to considerable clinical interest and changes in clinical practice.

## Acknowledgments

The authors thank the reviewers for their valuable comments on this study.

## Author contributions

**Funding acquisition:** Shuang Wu.

**Investigation:** Ligong Xu.

**Methodology:** Ligong Xu, Shuang Wu.

**Software:** Ligong Xu.

**Writing – original draft:** Ligong Xu, Shuang Wu.

**Writing – review & editing:** Ligong Xu, Shuang Wu.
